# Men’s Experiences and Decision-Making in Erectile Dysfunction Treatment: A Qualitative Evidence Synthesis

**DOI:** 10.7759/cureus.111413

**Published:** 2026-06-24

**Authors:** Anayat Ullah, Amer K Hussain, Taha Chaudhry

**Affiliations:** 1 Urology, Sandwell and West Birmingham Hospital NHS Trust, Birmingham, GBR; 2 Urology, Royal Cornwall Hospital Trust, Truro, GBR; 3 Urology, Dudley Group NHS Foundation Trust, Dudley, GBR

**Keywords:** erectile dysfunction, erectile dysfunction treatment, help-seeking, masculine identity, men’s sexual health, qualitative evidence synthesis, urology andrology erectile dysfunction

## Abstract

This paper presents a qualitative evidence synthesis investigating men’s clinical experiences, psychosocial challenges, and decision-making related to help-seeking and treatment for erectile dysfunction (ED). The findings suggest that ED affects relationship experiences, self-esteem, psychosocial well-being, and help-seeking behaviors. Studies identified through Scopus, PubMed, and PsycINFO were synthesized qualitatively, focusing on adult men’s experiences of ED, disclosure, treatment engagement, and decision-making. The review protocol was not prospectively registered. Searches were conducted in March 2026, and the included studies represented diverse clinical contexts, including general ED care, diabetes care, cancer survivorship, and cardiovascular rehabilitation. Eight qualitative studies involving 209 participants were included, and four major themes emerged: masculinity, stigma, and self-silencing; relational consequences influencing help-seeking and disclosure; healthcare communication as a barrier or facilitator to care; and treatment trade-offs, adherence, and acceptability. Men frequently reported negative effects on emotional well-being and help-seeking behaviors due to ED. Delay and hesitation in disclosure and help-seeking were attributed to fear of judgment, lack of support, and negative effects on confidence and self-esteem. Participants valued respectful communication from healthcare professionals and preferred supportive, individualized approaches that included relationship support. Decisions to seek treatment were influenced not only by perceived effectiveness but also by side effects, emotional burden, relationship concerns, and communication experiences. The findings should be interpreted with consideration of the limited number of included studies and the restriction to English-language publications. These findings suggest that ED care should incorporate respectful, stigma-sensitive communication and individualized counseling to facilitate treatment-seeking and improve treatment engagement.

## Introduction and background

Erectile dysfunction (ED) is associated with significant psychological, relational, and quality-of-life consequences. Contemporary clinical management includes lifestyle and risk-factor modification, counseling and sex therapy when indicated, oral phosphodiesterase type 5 inhibitors, vacuum erection devices, intracavernosal or intraurethral pharmacotherapy, and penile prosthesis implantation for selected patients [1]. Although multiple effective treatment options are available, men may hesitate to seek support or disengage from care [2,3]. This is partly because ED and related sexual health concerns are often stigmatized [3]. ED is also perceived to negatively affect masculine self-perception and self-esteem [4]. Qualitative research suggests that ED may be interpreted as a challenge to masculinity and self-esteem [5]. Rooted in broader masculinity norms, disclosure to partners and healthcare professionals is often limited due to embarrassment and fear of stigmatization [6].

These experiences shape men’s help-seeking behavior, disclosure, and treatment decision-making [6]. Such decisions include whether to seek professional care, adopt self-management strategies, or continue treatment [4,6]. Qualitative evidence on this topic is dispersed across diverse clinical contexts, including general ED care, cancer rehabilitation, cardiovascular disease, diabetes, and other healthcare settings [3]. To our knowledge, previous reviews have primarily focused on clinical management and treatment outcomes, whereas qualitative evidence synthesizing men’s experiences of ED, including help-seeking, disclosure, treatment engagement, and treatment-related decision-making across diverse clinical contexts, remains limited. This review addresses this gap by integrating qualitative findings to inform patient-centered communication and service design. A qualitative evidence synthesis can consolidate these findings to inform communication strategies and service design. This review aimed to synthesize qualitative evidence on men’s experiences of ED and the factors influencing decisions to seek support and select, initiate, or continue ED treatments.

## Review

Materials and methods

Design and Reporting

This qualitative evidence synthesis investigated men's psychosocial and clinical experiences related to help-seeking and treatment. The review also explored factors influencing help-seeking, treatment decision-making, and disclosure to partners and healthcare professionals. The review primarily focused on men's experiences across diverse clinical contexts and on the impact of psychosocial, relational, and treatment-related factors on treatment engagement. The SPIDER tool (Sample, Phenomenon of Interest, Design, Evaluation, Research type) was used to guide the review's structure, scope, and thematic focus [6]. The review protocol was not prospectively registered in PROSPERO or another review registry. Reporting of this qualitative evidence synthesis was guided by the Enhancing Transparency in Reporting the Synthesis of Qualitative Research (ENTREQ) statement [7]. The completed ENTREQ checklist is provided as Appendix A.

Eligibility Criteria

Primary research studies involving qualitative methods, including semi-structured interviews and/or focus groups, as well as mixed-methods studies with qualitative findings, were considered eligible for this qualitative evidence synthesis. Eligible studies involved adult men aged 18 years and older experiencing ED across diverse contexts and clinical settings. Studies were included if they reported men's experiences of ED and factors shaping treatment-seeking and engagement with treatment, disclosure, treatment perceptions, and other treatment experiences such as continuation, adherence, and acceptability. No minimum number of ED-specific participants or ED-specific interview questions was required; however, included studies needed to provide sufficient qualitative data related to men's experiences of ED, including its psychosocial, relational, or treatment-related impacts. Studies focusing primarily on other sexual health conditions not directly related to ED were excluded. Examples included studies focused exclusively on premature ejaculation, female sexual dysfunction, infertility, sexually transmitted infections, or general sexual health concerns without a specific focus on men’s experiences of ED, ED-related decision-making, or treatment engagement. Only studies published in English were included. This language restriction was applied due to feasibility considerations and is recognized as a potential source of selection bias, as relevant qualitative evidence published in other languages may not have been captured.

Information Sources and Literature Approach

Relevant literature was retrieved through database searches of PubMed/MEDLINE, Scopus, and PsycINFO in March 2026. The search was limited to studies published from January 2000 to March 2026 to capture contemporary evidence on men’s experiences of ED, treatment engagement, and decision-making. CINAHL was not included, which may represent a potential limitation of the search strategy. Search strategies combined terminology related to ED with other relevant terms such as qualitative methods, help-seeking, stigma, treatment perceptions, treatment experiences, and treatment decision-making. Search approaches were adapted to the indexing structure and syntax of each database. Reference lists of relevant studies were reviewed, and review articles were also used to identify additional relevant studies. Appendix B provides search terms and database strategies.

Study Selection

Studies were screened for relevance to the objectives of the review and eligibility criteria, primarily focusing on qualitative reporting of men's experiences related to ED help-seeking and treatment experiences. Titles and abstracts were independently screened by two reviewers against the predefined eligibility criteria. Full-text articles of potentially eligible studies were then independently assessed by the same reviewers. Any disagreements during screening or full-text assessment were resolved through discussion, and consensus was reached before final inclusion. Any disagreements that could not be resolved through discussion were adjudicated by a third reviewer.

Data Extraction

Bibliographic details such as author, year, and country were extracted for each included study, including study characteristics such as design, setting, participant characteristics, sample size, context, data collection methods, and findings related to self-perception, help-seeking, stigma, treatment experiences, and decision-making. For studies involving mixed populations, only qualitative data directly related to men’s experiences of ED, ED-related concerns, treatment decisions, disclosure, or help-seeking were extracted and included in the synthesis. In Gahm et al., although the study included 18 participants, extraction and analysis were limited to findings reported by the 10 participants with ED. Data from participants without ED were not included in the thematic synthesis [5].

Quality Appraisal

The methodological quality of included studies was assessed using the Critical Appraisal Skills Programme (CASP) qualitative checklist [8]. Quality appraisal was conducted by all the three authors using the predefined CASP criteria. The appraisal focused on study aims, methodology, recruitment, data collection, researcher-participant relationships, ethical considerations, data analysis, and clarity of findings. Any uncertainties regarding appraisal decisions were discussed by authors to achieve consensus.

Data Synthesis

**Figure 1:**
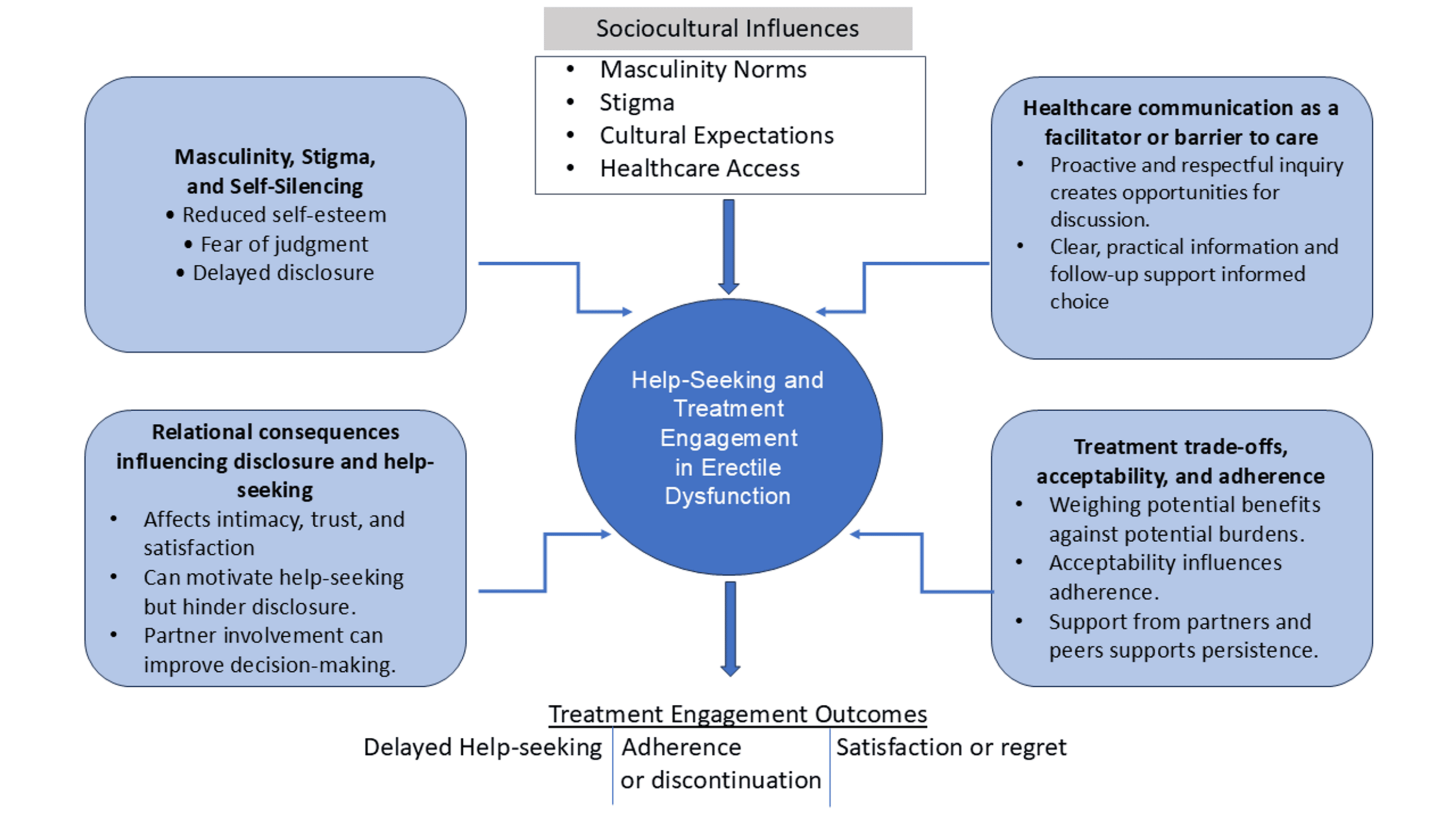
Thematic map of factors influencing help-seeking and treatment-related decision-making in erectile dysfunction Note: The map was derived from iterative coding, development of descriptive themes, and refinement of analytical themes following the approach of Thomas and Harden. Arrows represent conceptual relationships between themes and do not indicate causal pathways.

Qualitative findings were synthesized using the thematic synthesis approach described by Thomas and Harden [9]. The synthesis followed three iterative stages: (1) free line-by-line coding of findings reported in the included studies, (2) organization of related codes into descriptive themes that represented patterns across studies, and (3) development of analytical themes that extended beyond the primary study findings to address the review question and explain factors influencing men’s help-seeking, disclosure, treatment engagement, and decision-making. The final analytical themes were reviewed and refined through comparison across included studies. Coding and theme development were conducted manually using a structured approach based on the stages of thematic synthesis described by Thomas and Harden [9]. No qualitative data analysis software was used; instead, extracted findings and codes were organized systematically using structured data tables to support transparency and consistency during the synthesis process. Following the development of analytical themes, the relationships between themes and subthemes were visually mapped to illustrate the conceptual links identified during the synthesis process. Figure 1 was developed from the iterative coding process and represents the relationships between the analytical themes generated from the included studies rather than a post-hoc illustrative summary.

Results

Study Selection

**Figure 2:**
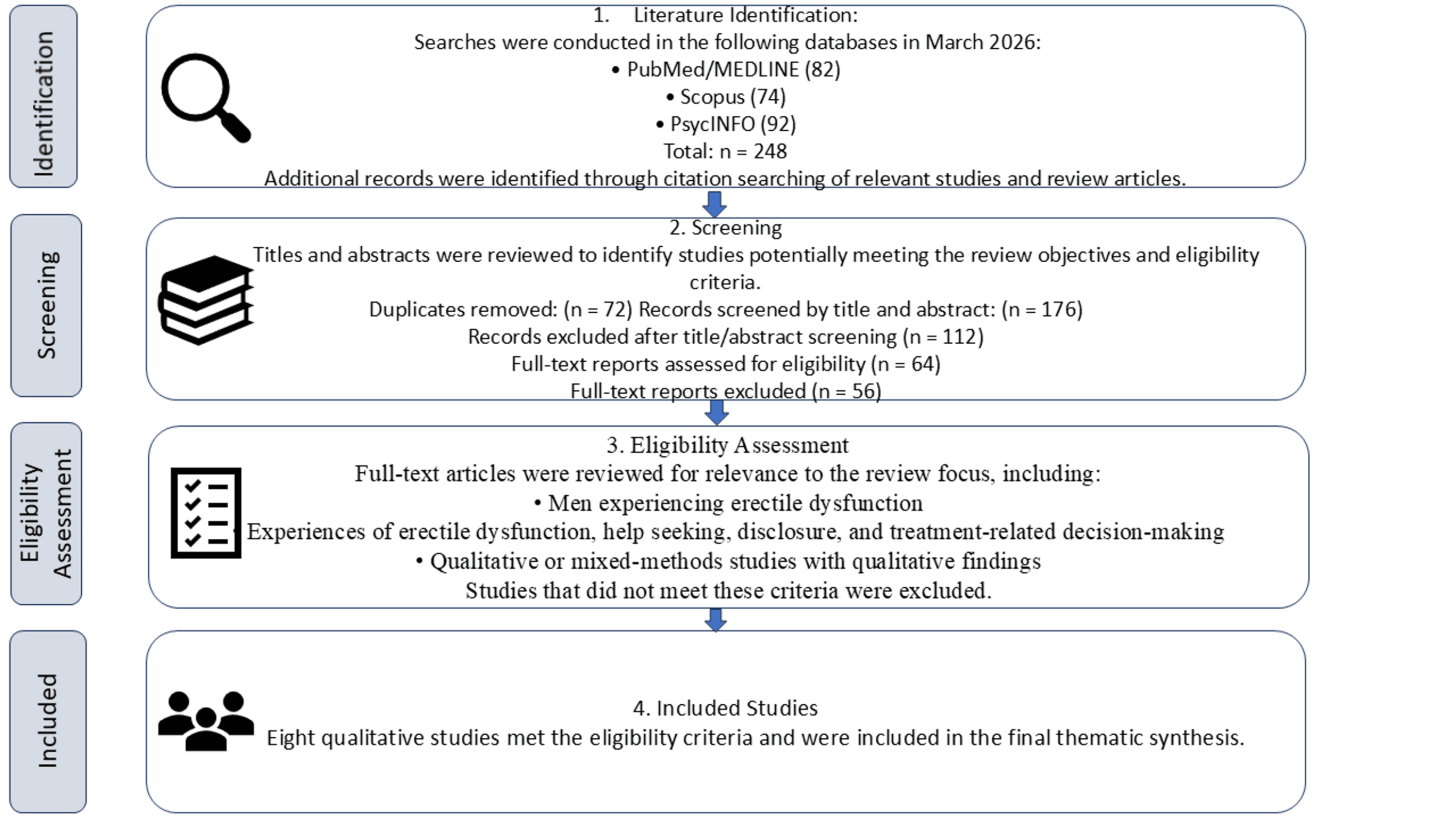
Overview of study identification and selection process

Relevant qualitative studies investigating men's experiences of ED across diverse contexts and clinical settings were identified through database searches and supplementary literature screening. Title and abstract screening and full-text assessment were independently conducted by all three reviewers using the predefined eligibility criteria. Any disagreements regarding study eligibility were resolved through discussion and consensus among the reviewers. Inter-rater agreement statistics were not calculated. The database search identified 248 records from PubMed/MEDLINE (n=82), Scopus (n=74), and PsycINFO (n=92). After removal of 72 duplicates, 176 records underwent title and abstract screening. Of these, 112 records were excluded, and 64 full-text articles were assessed for eligibility. Following full-text assessment, 56 articles were excluded for reasons including insufficient ED-specific qualitative data, inappropriate study design, and irrelevant populations or contexts. Eight studies met the inclusion criteria and were included in the qualitative evidence synthesis. Following screening and eligibility assessment, eight qualitative studies met the eligibility criteria and were included in the final synthesis, as shown in Figure 2.

Included Study Characteristics

Eight qualitative studies were included in the qualitative evidence synthesis. Studies were conducted in the United Kingdom, Denmark, Sweden, the United States, South Africa, and Malawi. These studies explored general ED care, primary care sexual health services, diabetes care, cancer survivorship, and cardiac rehabilitation. Across the included studies, data collection methods included interviews and focus groups, with a total of 209 men. The sample size across studies ranged from 13 to 47 participants. The study characteristics are summarized in Table 1.

**Table 1:** Characteristics of included qualitative studies ED, erectile dysfunction.

Study	Country	Design	Sample	Age (range/summary)	Setting/context	Data collection	Findings relevant to ED
Tomlinson and Wright, 2004 [2]	United Kingdom	Exploratory qualitative study using semi-structured interviews	40	22-72 years	Men attending a men’s health clinic for ED are prescribed sildenafil treatment	Semi-structured interviews	ED was associated with masculinity concerns and reduced self-esteem. Successful treatment was associated with improved confidence and emotional well-being, whereas unsuccessful treatment often intensified disappointment and distress.
Dowswell et al., 2011 [3]	United Kingdom	Qualitative interviews with framework analysis	28	34-80 years	Men treated for colorectal cancer with sexual dysfunction, including ED	Semi-structured face-to-face interviews	Help-seeking was influenced by embarrassment, information gaps, unclear referral pathways, and perceived age-related dismissal by clinicians. Men valued proactive information and follow-up regarding sexual health concerns.
Ball et al., 2013 [10]	United States	Qualitative interviews and focus groups	13	47-82 years	Rectal cancer survivorship and sexual dysfunction, including ED	Telephone interviews and in-person focus groups	Men often prioritized cancer survival over sexual function during early recovery, but later desired clearer information regarding sexual side effects and treatment options. Embarrassment and practical barriers reduced participation in sexual health interventions.
Nelson et al., 2015 [11]	United States	Focus groups with thematic analysis	30	41-72 years	Penile rehabilitation following radical prostatectomy	Focus groups	Emotional distress and avoidance affected adherence to penile rehabilitation. Injection therapy was sometimes perceived as uncomfortable or unnatural. Partner and peer support contributed to persistence with rehabilitation efforts.
Cooper et al., 2018 [4]	South Africa and Malawi	Interviews and focus groups with thematic analysis	47	28-78 years	Men with type 2 diabetes discussing sexual well-being, including ED	Interviews and focus group discussions	Sexual difficulties affected self-perception, intimacy, and relationship communication. Men described fear of judgment and negative healthcare encounters. Cost and limited access influenced treatment decisions, while silence within relationships was common.
Gerbild et al., 2021 [12]	Denmark	Semi-structured interviews with content analysis using an acceptability framework	20	48-78 years	Cardiac rehabilitation and communication regarding ED and sexuality	Individual interviews	Men generally accepted healthcare professionals' initiation of discussion about ED when approached respectfully and competently. Sexuality was often perceived as a sensitive or taboo topic, and participants preferred private, individualized counseling.
Gahm et al., 2024 [5]	Sweden	Semi-structured interviews with qualitative content analysis	18 total participants; 10 with ED	Adults aged 20 years or older	Primary care sexual health clinic population	Telephone interviews	Men sought care to exclude physical illness and to access medication or counseling. Shame and fear of inadequacy contributed to avoidance of care. ED frequently affected relationship dynamics and influenced decisions regarding disclosure and help-seeking.
Hansen et al., 2025 [13]	Denmark	Semi-structured interviews with content analysis	13	48-78 years	Men undergoing rectal cancer surgery with preoperative ED education focus	Telephone interviews	Men valued direct and practical information regarding postoperative sexual outcomes and often preferred partner involvement in discussions. Some participants maintained optimistic expectations despite limited discussion of postoperative sexual consequences.

Quality Appraisal

The selected studies demonstrated clearly defined qualitative research aims, appropriate qualitative designs, and coherent reporting of findings. Common limitations included limited reporting of reflexivity and inconsistent reporting of participant recruitment strategies and transferability. Methodological appraisal was conducted using the CASP qualitative checklist [8]. CASP findings were not converted into numerical scores or used for exclusion decisions. Instead, an overall narrative judgment was used to summarize the methodological quality of each study. Studies described as “Strong” demonstrated clear alignment between the research aim, qualitative methodology, data collection, analysis, and reporting of findings, with minimal methodological concerns identified. Studies described as “Generally strong” demonstrated appropriate methodological approaches but had some limitations in areas such as reflexivity, reporting of researcher-participant relationships, ethical considerations, or analytic transparency. In line with the recommended use of the CASP framework, appraisal findings were interpreted narratively rather than through quantitative or numerical scoring. Table 2 provides a quality appraisal summary of the selected studies.

**Table 2:** Quality appraisal summary of included studies using the CASP qualitative checklist Note: The categories “Strong” and “Generally strong” represent narrative summaries of methodological quality based on CASP domains and are not formal quality scores. CASP, Critical Appraisal Skills Programme; ED, erectile dysfunction.

Study	Overall appraisal	Key strengths	Major limitations affecting confidence
Tomlinson and Wright, 2004 [2]	Strong methodological quality	Clear study aims, appropriate interview methods, and a clear relationship between participant accounts and interpretation	Limited reporting of reflexivity
Dowswell et al., 2011 [3]	Strong methodological quality	Purposive sampling, clear framework analysis approach, and strong contextual detail	Reflexivity reporting was limited. Additional limitations included limited discussion of ethical considerations.
Ball et al., 2013 [10]	Generally strong methodological quality	Use of both interviews and focus groups, with a clear description of thematic analysis	Small and selective sample with limited participant diversity
Nelson et al., 2015 [11]	Strong methodological quality	Clear qualitative methods, sampling approach, theme development, and use of illustrative quotations	The focus group format may have limited disclosure of sensitive experiences.
Cooper et al., 2018 [4]	Strong methodological quality	Multi-site qualitative design, clear theme development, and ethical considerations appropriately addressed	Sexual well-being was not the sole focus of the study, which may have limited the depth of ED-specific treatment experiences.
Gerbild et al., 2021 [12]	Generally strong methodological quality	Theory-informed acceptability analysis and clear participant descriptions	Findings were specific to cardiac rehabilitation settings and not directly relevant to sexual health and ED.
Gahm et al., 2024 [5]	Strong methodological quality	Clear reporting of themes related to help-seeking, relationships, and treatment perceptions	Inclusion of participants with both ED and premature ejaculation required careful extraction of ED-specific findings.
Hansen et al., 2025 [13]	Strong methodological quality	Clear reporting of interview methods, coding, and qualitative analysis	Single-center context may limit transferability; member checking was not reported.

Thematic Synthesis

Four analytic themes emerged from men’s experiences and treatment-related decision-making across diverse clinical contexts. These themes included masculinity, stigma, and self-silencing; relational consequences influencing help-seeking and disclosure; healthcare communication as a barrier or facilitator to care; and treatment trade-offs, adherence, and acceptability.

Masculinity, Stigma, and Self-Silencing

Across diverse clinical contexts, men associated ED with reduced self-esteem and challenges to masculinity [2,11]. Feelings of embarrassment and perceived stigma contributed to limited disclosure, hesitation to seek help, and disengagement from treatment [2,5,10]. Sexual health concerns were often perceived as taboo topics in primary care and chronic disease settings [4,13]. Participants also described fear of judgment from partners and healthcare professionals, contributing to limited disclosure and hesitation during treatment decision-making [3,12].

Relational Consequences Influencing Disclosure and Help-Seeking

ED negatively affected intimacy, communication, and emotional connection within relationships [3,12]. These concerns sometimes encouraged men to seek treatment. However, fear of disappointing partners and concerns regarding relationship strain also contributed to limited disclosure and hesitation in seeking care [4,5,11]. In cancer survivorship settings, participants valued clear information regarding the sexual side effects of treatment and preferred partner involvement during consultations to support shared decision-making [2,10,13].

Healthcare Communication as a Facilitator or Barrier to Care

Men’s engagement with treatment was strongly influenced by communication with healthcare professionals [2,5]. Participants valued respectful and supportive communication, as well as proactive discussions involving individualized counseling and follow-up support [3,12,13]. Conversely, dismissive attitudes, assumptions regarding sexual activity, and negative treatment perceptions contributed to hesitation and delayed help-seeking [4,10,11].

Treatment Trade-Offs, Acceptability, and Adherence

Treatment decisions reflected a balance between perceived benefits and perceived burdens [3,11]. Perceived benefits included improvements in intimacy, confidence, and relationship satisfaction, whereas perceived burdens included financial costs, side effects, invasiveness, and embarrassment [2,5]. In penile rehabilitation settings, structured rehabilitation regimens such as injections and medications were associated with reduced adherence compared with more individualized care approaches [4,10]. Support from partners and healthcare professionals, together with open discussion of treatment experiences, was associated with continued treatment engagement [12,13].

Across the included studies, these interconnected factors influenced several downstream experiences related to ED care. Stigma, concerns regarding masculinity, and communication barriers contributed to delayed help-seeking and limited disclosure. Treatment decisions were shaped by perceived benefits, burdens, and individual circumstances, influencing continued engagement, adherence, or discontinuation of treatment. Experiences with treatment outcomes, communication, and relationship impacts also shaped perceptions of satisfaction, acceptance, or regret regarding treatment choices.

Figure 1 illustrates relationships among masculinity, stigma, and self-silencing; relational consequences influencing disclosure and help-seeking; healthcare communication as a facilitator or barrier to care; treatment trade-offs, acceptability, and adherence; and treatment engagement outcomes in ED.

Discussion

Principal Findings

This qualitative evidence synthesis suggests that men’s ED treatment decisions are shaped not only by perceived treatment efficacy but also by an interplay among masculinity-related concerns, stigma, relational dynamics, healthcare communication, and practical treatment burdens. Men frequently described hesitation in seeking treatment due to embarrassment, fear of stigmatization, and discomfort discussing sexual health concerns. Conversely, respectful and proactive communication from healthcare professionals appeared to support treatment engagement and help-seeking behaviors.

Comparison With Existing Literature and Theoretical Perspectives

The findings of this qualitative evidence synthesis are consistent with broader literature on men’s health behaviors, particularly research examining how masculinity norms influence healthcare engagement and help-seeking. Theoretical perspectives on masculinity and health behavior, including Courtenay’s work, suggest that socially constructed expectations of masculinity may encourage men to minimize vulnerability, maintain self-reliance, and delay seeking healthcare support. The present findings extend this understanding by demonstrating how these processes operate within the specific context of ED, where symptoms may be closely linked with perceptions of sexual identity, competence, and relational expectations.

Previous literature on men’s help-seeking has highlighted that stigma, emotional restraint, and concerns regarding social judgment can contribute to delayed engagement with healthcare services. This synthesis supports these observations but further demonstrates that ED represents a particularly sensitive health concern because it involves both physical symptoms and meanings associated with masculinity, intimacy, and partnership. Therefore, decisions regarding disclosure and treatment are not based solely on symptom severity or treatment effectiveness but are shaped by broader psychosocial and relational considerations.

The findings also extend existing understandings of treatment engagement by emphasizing that men evaluate ED interventions through experiences of acceptability, communication, and personal meaning. While biomedical approaches have traditionally emphasized treatment efficacy, the qualitative evidence synthesized here suggests that supportive communication, individualized counseling, and attention to relationship context are essential components of care. These findings align with broader calls within men's health research to move beyond models that view men’s healthcare decisions as individual choices and instead consider the social and cultural contexts influencing those decisions.

Contextual Variation Across Clinical Settings

Although common patterns were identified across the included studies, the findings should be interpreted within the clinical and cultural contexts in which ED was experienced. Men’s perceptions of ED and decisions regarding disclosure or treatment may vary depending on the underlying health condition, healthcare setting, and relationship to illness. For example, studies conducted in cancer survivorship contexts highlighted concerns related to treatment-related sexual changes, rehabilitation expectations, and the importance of partner communication, whereas studies involving chronic conditions such as diabetes and cardiovascular disease emphasized the interaction between ED, broader health management, and healthcare engagement.

Differences across settings may also reflect variation in cultural expectations surrounding masculinity, sexuality, and discussions of sexual health. In contexts where sexual concerns remain highly stigmatized, men may experience greater hesitation in seeking support or disclosing symptoms. Conversely, clinical environments that normalize sexual health discussions and provide structured opportunities for communication may facilitate earlier engagement with care. Therefore, while the synthesized themes represent recurring patterns across studies, their expression may differ according to cultural norms, clinical context, and individual circumstances.

Implications for Clinical Practice and Service Design

Findings from this qualitative evidence synthesis support ED care approaches that normalize sexual health discussions and reduce stigma associated with treatment-seeking. In diabetes care, cancer survivorship, and cardiovascular rehabilitation settings, participants valued individualized communication, clear information, and ongoing follow-up support. Counseling approaches may benefit from addressing concerns related to relationships, side effects, embarrassment, and treatment expectations to support informed decision-making. Several studies also suggested that partner involvement during consultations may improve communication and treatment engagement when desired by patients [3,11,13]. These findings further suggest that treatment frameworks perceived as effective but insufficiently supportive or individualized may negatively affect treatment engagement and overall well-being [11].

Future Research

Future research should address several specific gaps identified in this synthesis. First, more qualitative studies are needed among culturally diverse populations and groups that remain underrepresented in the existing literature, including men from different geographic regions, ethnic backgrounds, and varied sexual orientations. Such research may clarify how cultural expectations, masculinity norms, and social contexts influence ED disclosure and treatment-seeking behaviors.

Second, additional research is needed to examine experiences across specific clinical contexts and treatment pathways. While included studies addressed settings such as cancer survivorship, diabetes, cardiovascular disease, and general ED care, further investigation is needed to understand how men experience treatment decisions within different healthcare systems and disease contexts. Studies exploring experiences with specific interventions, including pharmacological treatments, rehabilitation approaches, and surgical options, may provide greater insight into treatment acceptability, adherence, and discontinuation.

Third, future studies should examine the perspectives of partners and healthcare professionals, as ED-related decision-making often occurs within relational and communication contexts. Research exploring interventions that improve clinician-patient communication, reduce stigma, and support shared decision-making may help identify effective strategies for improving treatment engagement.

Finally, future qualitative research should prioritize transparent reporting of reflexivity, recruitment processes, and analytic procedures to strengthen methodological rigor and improve transferability of findings across settings.

Limitations of this Review

The included studies represented diverse cultural and clinical contexts, and the identified themes reflect patterns across these settings rather than a single uniform experience. Restriction to English-language publications may have excluded culturally diverse perspectives and limited the transferability of findings. In addition, this review depended on the reporting quality of the included primary studies, particularly with respect to recruitment transparency and reflexivity. Additionally, the review protocol was not prospectively registered, which may limit methodological transparency compared with prospectively registered evidence syntheses. The search strategy was limited to PubMed, Scopus, and PsycINFO and did not include databases such as CINAHL, which indexes nursing and allied health qualitative research. Therefore, potentially relevant studies published in nursing or broader healthcare contexts may not have been identified. Additionally, publication bias and reporting bias cannot be excluded because this review focused on published peer-reviewed literature and did not include gray literature sources such as dissertations, conference proceedings, or unpublished qualitative studies. Therefore, potentially relevant experiences that were not published in academic databases may not have been captured.

## Conclusions

Men’s experiences of ED extended beyond physical symptoms and were closely connected to self-esteem, masculinity-related concerns, relationship dynamics, and interactions with healthcare professionals. Across different clinical contexts, feelings of embarrassment, stigma, and fear of judgment frequently contributed to delayed help-seeking, limited disclosure, and uncertainty regarding treatment decisions. At the same time, supportive relationships, clear communication, and respectful clinical discussions could facilitate treatment engagement and improve confidence in decision-making.

This qualitative evidence synthesis highlights the importance of patient-centered approaches in ED care. Men valued direct yet sensitive communication, practical information about treatment options and side effects, and opportunities for individualized discussion in a private, supportive environment. Consideration of psychosocial and relational factors alongside treatment effectiveness may help improve treatment acceptability, adherence, and overall engagement with care among men experiencing ED.

## Appendices

Appendix A

ENTREQ Reporting Checklist for Qualitative Evidence Synthesis

**Table 3:** ENTREQ reporting checklist for qualitative evidence synthesis ENTREQ, Enhancing Transparency in Reporting the Synthesis of Qualitative Research.

No.	Item	Description	Reported in manuscript
1	Aim	State the aim of the qualitative evidence synthesis	Yes. The review's aim is described in the Introduction and Methods sections.
2	Synthesis methodology	Describe the methodology used for qualitative evidence synthesis	Yes. A qualitative evidence synthesis approach was used.
3	Approach to searching	Describe information sources and search strategy	Yes. Searches were conducted in PubMed/MEDLINE, Scopus, and PsycINFO in March 2026. Search strategies are provided in Appendix B.
4	Inclusion criteria	Describe eligibility criteria for included studies	Yes. Eligibility criteria are described in the Methods section.
5	Study selection	Describe the process used to select studies	Yes. Study identification, screening, eligibility assessment, and inclusion are described and summarized in Figure 1.
6	Study characteristics	Describe characteristics of included studies	Yes. Study characteristics are presented in Table 1.
7	Quality appraisal	Describe methods used to assess study quality	Yes. Study quality was appraised using the Critical Appraisal Skills Programme (CASP) qualitative checklist.
8	Data extraction	Describe methods used to extract relevant data	Yes. Bibliographic details, study characteristics, and findings relevant to the review objectives were extracted.
9	Data synthesis	Describe methods used to synthesize qualitative findings	Yes. Findings were synthesized through iterative review and thematic coding.
10	Researcher characteristics and reflexivity	Describe researcher influence and reflexivity considerations	Limited reporting available from included primary studies; this limitation is acknowledged in the manuscript.
11	Study findings	Present synthesized findings and supporting themes	Yes. Four analytical themes are presented in the Results section.
12	Transparency of synthesis	Provide sufficient detail to understand how interpretations were developed	Yes. The thematic synthesis process and relationship between themes are described.
13	Limitations	Describe limitations of the synthesis	Yes. Limitations related to study number, language restriction, and reporting quality are discussed.
14	Implications	Describe implications for practice and future research	Yes. Clinical and research implications are discussed in the Discussion section.

Appendix B

Search Strategies

PubMed/MEDLINE:

("erectile dysfunction"[Mesh] OR "erectile dysfunction"[tiab] OR ED[tiab] OR "sexual dysfunction"[tiab])

AND ("qualitative"[tiab] OR "interviews"[tiab] OR "focus groups"[tiab] OR "thematic"[tiab])

AND ("help-seeking"[tiab] OR "decision-making"[tiab] OR "treatment"[tiab] OR "experience"[tiab])

Limits: English language; human subjects.

Scopus

TITLE-ABS-KEY( ("erectile dysfunction" OR ED OR "sexual dysfunction") AND (qualitative OR interviews OR "focus groups" OR thematic) AND ("help-seeking" OR "decision-making" OR treatment OR experience) ) AND LANGUAGE(english)

PsycINFO

( DE "Erectile Dysfunction" OR TI("erectile dysfunction" OR ED OR "sexual dysfunction") OR AB("erectile dysfunction" OR ED OR "sexual dysfunction") ) AND ( TI(qualitative OR interviews OR "focus groups" OR thematic) OR AB(qualitative OR interviews OR "focus groups" OR thematic) ) AND ( TI("help-seeking" OR "decision-making" OR treatment OR experience) OR AB("help-seeking" OR "decision-making" OR treatment OR experience) ) Limiters: English language; human participants.
